# Response to “two cases of ulcerative dermatomyositis with negative antimelanoma differentiation-associated gene 5 antibody”

**DOI:** 10.1016/j.jdcr.2025.03.014

**Published:** 2025-03-29

**Authors:** Mehmet Fatih Atak, Banu Farabi, Shoshana Marmon

**Affiliations:** a Dermatology Department, New York Medical College, Valhalla, New York; b Dermatology Department, NYC H+H/Metropolitan, New York, New York; c Department of Medicine, NYC H+H/South Brooklyn, New York, New York

**Keywords:** anti-MDA5 antibodies, dermatomyositis, ulceration

*To the Editor:* We read with great interest the letter by Choi et al, describing 2 additional cases of ulcerative dermatomyositis (DM) in the absence of anti-melanoma differentiation-associated gene 5 (MDA)-5 antibodies. Their findings build upon our previously published case of treatment-refractory, anti-MDA-5-negative DM with recurrent cutaneous ulcerations, which ultimately responded favorably to mycophenolate mofetil.^1^

Cutaneous ulceration is an uncommon but significant feature of DM, affecting approximately 3% to 19% of patients.^2^ These ulcers are often painful, debilitating, and challenging to manage, contributing to substantial morbidity.^2^^,^^3^ Anti-MDA-5 antibodies are strongly associated with ulceration in DM, and this combination is linked to an increased risk of rapidly progressive interstitial lung disease (ILD).^2^ The systemic vasculopathy observed in anti-MDA-5-positive DM has been proposed as a key factor in the connection between cutaneous ulcers and ILD, and skin biopsies from ulcerated lesions in these patients frequently reveal vasculopathic changes.^2^^,^^3^

Ulceration in DM without anti-MDA-5 antibodies, as observed in our case and the 2 reported by Choi et al, is less common and remains poorly defined. Unlike the vasculopathic findings typically seen in anti-MDA-5-positive DM, skin biopsies from our patients revealed subtle interface dermatitis compatible with a manifestation of collagen vascular disease. Cases of ulcerative DM without MDA-5 antibodies identified in the literature appear to support a similar clinical profile marked by refractory ulcerations and the absence of vasculopathy on biopsy (Table I). Among these patients, the occurrence of ILD, malignancy, and other serologic markers was more variable.

**Table I:** Clinical and serologic features of ulcerative dermatomyositis in patients without anti-MDA-5 antibodies

Author(s)	Tomb et al, 2002^4^	Al Awqati et al, 2016^5^	Ozer et al, 2024^1^	De Jesus et al, 2024^6^	Wald et al, 2025 Case 1^7^	Wald et al, 2025 Case 2^7^	Choi et al, 2025 Case 1	Choi et al, 2025 Case 2
Demographics	34 F	60 F	53 F	52 F	33 F	67 F	37 M	84 M
Vasculopathy on biopsy	No vasculopathy on biopsies	Not specified	No vasculopathy on biopsies	Not specified	Not specified	Not specified	No vasculopathy on biopsies	No vasculopathy on biopsies
Muscle involvement	Typical with myositis?	Myositis present with EMG	Clinically amyopathic with elevated CK levels	Clinically amyopathic but fulfill myositis criteria	Myositis with elevated CK	Fluctuant CK	Normal CK	Normal CK
Presence of ILD	Unknown	No	No	Yes	No	No	Yes	No
Anti-MDA-5	Unknown	Negative	Negative	Negative	Negative	Negative	Negative	Negative
ANA	Unknown	N/A	Positive	Positive	Negative	Positive	Negative	Negative
Anti-RO52	Unknown	N/A	Positive	Negative	N/A	N/A	Positive	N/A
Antibeta-2 glycoprotein	Unknown	N/A	Positive	N/A	N/A	N/A	N/A	N/A
Anti-MI-2 antibodies	Unknown	N/A	Negative	N/A	N/A	N/A	N/A	N/A
TIF1-y	Unknown	Positive	Negative	Negative	N/A	Positive	N/A	N/A
Anti-NXP2 (nuclear matrix protein 2)	Unknown	N/A	Negative	Negative	Positive	N/A	N/A	N/A
Anti-Jo	Unknown	N/A	Negative	N/A	N/A	N/A	N/A	N/A
Malignancy	Unknown	Ovarian cancer	No	No	No	Bronchial carcinoma	No	No
Refractory to	Refractory oral steroids	N/A	IV pulse steroids and IM methotrexate	Oral steroids	N/A	N/A	N/A	N/A
Responsive to	Refractory to oral steroids and azathioprine	IVIG, high-dose pulse corticosteroids	Mycophenolate mofetil	IV pulse steroids	IVIG, methylprednisolone pulse therapies, azathioprine	Intravenous iloprost and bosentan, IVIG, oral steroids	N/A	N/A

*ANA*, Anti-nuclear antibody; *CK*, creatinin kinase; *EMG*, electromyography; *F*, female; *ILD*, interstitial lung disease; *IM*, intramuscular; *IVIG*, intravenous immunoglobulin; *IV*, intravenous; *M*, male; *MDA-5*, melanoma differentiation-associated gene 5 antibody; *N/A*, not applicable.

Further research is needed to determine whether this subtype of DM has distinct pathogenic and prognostic implications. We appreciate the contributions of Choi et al^8^ in expanding the discussion on this less common presentation of DM and support continued investigation into its underlying mechanisms and optimal management.

## Conflicts of interest

None disclosed.
